# The Framing Effect of Digital Textual Messages on Uptake Rates of Medical Checkups: Field Study

**DOI:** 10.2196/45379

**Published:** 2024-03-06

**Authors:** Amnon Maltz, Stella Rashkovich, Adi Sarid, Yafit Cohen, Tamar Landau, Elina Saifer, Neta Amorai Belkin, Tamar Alcalay

**Affiliations:** 1 Department of Economics University of Haifa Haifa Israel; 2 Nursing Division Maccabi Healthcare Services Tel Aviv Israel; 3 Sarid Research Services Haifa Israel; 4 Marketing Automation Department Maccbi Healthcare Services Tel Aviv Israel; 5 AI & Big Data Department Maccabi Healthcare Services Tel Aviv Israel

**Keywords:** field experiment, nudge, cancer screening, mammography, vaccination, framing, fecal occult blood test (FOBT), behavior change

## Abstract

**Background:**

Health care authorities often use text messages to enhance compliance with medical recommendations. The effectiveness of different message framings has been studied extensively over the past 3 decades. Recently, health care providers have begun using digital media platforms to disseminate health-related messages.

**Objective:**

This study aimed to examine the effectiveness of some of the most widely used message framings on the uptake rates of medical checkups.

**Methods:**

This study used a large-scale digital outreach campaign conducted by Maccabi Healthcare Services (MHS) during 2020-2021, involving a total of 113,048 participants. MHS members aged 50-74 years were invited to take their recommended medical actions from the following list: human papillomavirus (HPV), mammography, abdominal aortic aneurysm, fecal occult blood test (FOBT), and pneumococcal vaccination. Each member was randomly assigned to receive 1 of 6 message framings: control (neutrally framed; n=20,959, 18.5%), gains (benefits of compliance; n=20,393, 18%), losses (negative consequences of noncompliance; n=15,165, 13.4%), recommendation (a recommendation by an authoritative figure, in this context by a physician; n=20,584, 18.2%), implementation intentions (linking potential outcomes to future reactions; n=20,701, 18.3%), and empowerment (emphasizing personal responsibility for maintaining good health; n=15,246, 13.5%). The time frames for measuring a successful intervention were 14 days for scheduling screenings (ie, HPV, mammography, or abdominal aortic aneurysm), 30 days for performing the FOBT, and 60 days for receiving pneumococcal vaccination. We also examined the effectiveness of media channels (text message or email) on uptake rates and whether the subject-line length is correlated with message-opening rates.

**Results:**

No significant effect of message framing on uptake rates of medical checkups was observed. The rates of appointments for screening ranged from 12.9% to 14.1% across treatments. Based on a chi-square test, there was no evidence to reject the null hypothesis that these compliance rates are independent of the treatments (*P*=.35). The uptake rates for the FOBT and pneumococcal vaccination ranged from 23.3% to 23.8% across treatments, and we could not reject the hypothesis that they are independent of the treatments (*P*=.88). We also found that emails are more effective than text messages (*P*<.001) and that the subject-line length is negatively correlated with message-opening rates.

**Conclusions:**

No evidence was found for an effect of the 5 message framings on uptake rates of medical checkups. To enhance compliance rates, public health officials may consider alternative framings. Furthermore, media channels and the subject-line length should be given careful consideration in the planning stages of health care campaigns.

**Trial Registration:**

AEA RCT Registry AEARCTR-0006317; https://www.socialscienceregistry.org/trials/6317/history/201365

## Introduction

Successful preventive care requires high uptake rates of early detection tests. Increasing these rates at relatively low costs is one of the more pressing challenges faced by public health officials. Over the past 3 decades, behavioral insights have been used to nudge individuals and increase their compliance rates with recommendations of their national health care authorities. One common tool used in this line of research is *message framing*, that is, the content of textual messages sent out by health care providers. Message framing has been used to encourage healthy behavior in various public health contexts, such as smoking cessation [1-3], early-detection cancer screenings [4,5] influenza vaccinations [6-8], and, recently, COVID-19 vaccinations [9-11].

In this large-scale field study, we reexamined some of the most influential and commonly used message framings to shed light on their effectiveness. Specifically, we investigated prospect theory, physician’s recommendations, implementation intentions, and empowerment.

Prospect theory [12-14] incorporates the idea that losses loom larger than gains. Over the past 3 decades, dozens of studies have examined the effect of these types of framings on different medical outcomes. Results have been rather mixed and have led to an important ongoing discussion regarding the usefulness of prospect theory–based frames in medical contexts (see Refs. [5,15,16] for systematic reviews).

Physician’s recommendations have also been examined in the literature, especially with respect to cancer screenings, and have been found to increase uptake rates [17-19].

Implementation intentions [20,21] link potential outcomes (eg, results of medical tests) and reactions (eg, steps to be taken after results are received). Such links in critical situations have been shown to facilitate individuals in reaching their health goals [22]. Implementation intentions have been used in different medical contexts, such as influenza vaccination [7,23], colorectal cancer screenings, and the fecal occult blood test (FOBT), for which some studies have reported positive effects [24,25], while others have found no effect at all [26].

Empowerment messaging is meant to emphasize the individual’s responsibility to take care of their own health. Thus far, it has shown potential to increase breast and cervical cancer screening rates [27-29].

Our study contributes to this literature by examining the large-scale effectiveness of these types of framings. To do so, we made use of a massive digital outreach campaign that was held during 2020-2021 by Maccabi Healthcare Services (MHS), the second-largest health maintenance organization (HMO) in Israel.

In addition to the examination of message framing, we also investigated 2 other topics: the effect of the digital media channel on message-opening rates and the influence of the subject-line length on responsiveness. As health care organizations turn to digital media as their main means of communication with the public, there is growing interest in the role of different media channels in generating positive health outcomes [30-36] and in the impact of the subject-line length on digital marketing campaigns [37-39]. These topics are essential for enhancing the public’s responsiveness to health-related digital communication, and they were addressed in this study.

Although the digital setup of this study allowed us to shed light on these 2 topics of emerging interest in public health, the main aim of our work was to test whether specific types of framings can increase adherence to medical recommendations. More specifically, we tested the null hypothesis: the well-known types of framings of digital messages used in this study have no effect on the uptake rates of medical checkups.

## Methods

### Study Design

MHS contacted members aged 50-74 years and invited them to take preventive medical actions according to their age, medical history, and guidelines of the Israeli Ministry of Health. The campaign ran from July 2020 to December 2021 and targeted the following medical procedures: mammography (for women), human papillomavirus (HPV; for women), abdominal aortic aneurysm screening (for men), and the FOBT and pneumococcal vaccination (for men and women).

MHS members included in this campaign were randomly assigned into 1 of 6 groups:

- Control: received an informative message, which was identical to the one used by MHS prior to this study
- Gains: highlighting the benefits of compliance
- Losses: highlighting the potential negative effects of noncompliance
- Recommendation: citing a recommendation by an MHS physician
- Implementation intentions: stating a plan that links outcomes to reactions
- Empowerment: emphasizing the personal responsibility of the members for their own health

We included all members who received a digital message from MHS for the first time. Those who received a digital message prior to the initiation of the study were excluded. (MHS started sending out digital messages about 1 year before the initiation of the study as part of a large digitization process within the organization. These messages used the neutral frame that later became the control group in our study.)

Messages were communicated to members via email unless they lacked a valid email address in the MHS registry, in which case, a text message (SMS) was sent with a link redirecting to a landing page. The email subject line and contents (or the text message and the corresponding landing page) had 6 variations according to the assignment to control or treatment groups. Exact message contents are available in Multimedia Appendix 1.

Members received an invitation to perform 1 (or more) of 5 medical checkups that were recommended for them. These recommendations were based on the following criteria that combine the guidelines of the Israeli Ministry of Health with MHS’s designated target groups of the digital campaign:

- Mammography: women above the age of 50 years for whom at least 2 years had passed since their last mammography test
- HPV: women aged 50-54 years for whom at least 3 years had passed since their last HPV test
- FOBT: women and men above the age of 50 years for whom at least 1 year had passed since their last FOBT and at least 5-10 years had passed since their last colonoscopy test (the exact number of years since the last colonoscopy that triggers an invitation to perform the FOBT determined by the individual’s specific health risks)
- Pneumococcal vaccination: women and men above the age of 65 years who never received the vaccination
- Abdominal aortic aneurysm screening: men above the age of 65 years with a history of smoking who had never performed the screening

Note that members could have been eligible for more than 1 checkup. In these cases, the invitation message included all the relevant checkups.

### Procedure

Members who met 1 or more of the aforementioned criteria received a message from MHS in which they were invited to perform all the medical checkups that were recommended for them by the Israeli Ministry of Health. (Members who had already been diagnosed with a specific condition that 1 of the 5 medical procedures was meant to detect did not receive an invitation to test for that specific condition. They were contacted by MHS separately to follow up on their earlier diagnosis and to assess their overall medical well-being.) Following the initial contact, if the member scheduled an appointment or performed the recommended checkups, they did not receive any further messages. If they did not comply with the recommendations for all checkups that appeared on their initial message, they received reminders that followed the same framing theme that appeared in their initial message and included the remaining recommended checkups that the member did not perform or did not schedule an appointment for since the initial message was sent out. The first reminder was sent out 2 days after the first message but only in case the first message was sent via email and was not opened. The second and third reminders were sent out 2 and 4 weeks after the initial message, respectively (+/– a day or two if the reminder was supposed to be sent out on the weekend).

For mammography, HPV, and abdominal aortic aneurysm (which we call “screenings”), we collected data regarding scheduled appointments (we did not have access to the actual performance data of these screenings). For the FOBT and pneumococcal vaccination (which we call “lab tests”), we collected data regarding the actual performance (these procedures do not require appointments, and one can perform them simply by showing up at the clinic). Next to every medical procedure that appeared on a member’s invitation was a link that, once clicked, redirected the member to receive more information about the specific procedure on the website of MHS. Messages with screening recommendations included a link through which members could make the appointment (lab tests require no appointment). A total of 129,070 MHS members participated in this study, of which 113,048 (87.6%) were included in our analysis (the data-cleaning process is described in detail in Multimedia Appendix 2). Of these, 71,140 (62.9%) members received messages that only included lab tests, 30,878 (27.3%) received messages that only included screenings, and 11,030 (9.8%) received messages that included at least 1 lab test and at least 1 screening. Looking at the number of checkups that were included in the first invitation message of members who participated in the analysis, we found that 100,125 (88.6%) received a message that referred to only 1 recommended checkup, 10,416 (9.2%) received a message that included 2 recommended checkups, and 2507 (2.2%) received a message with 3 recommended checkups.

### Outcome Variables

We analyzed the data separately for screenings and for lab tests since each of the 2 has a different outcome measure (appointment scheduling vs actual performance, respectively). In addition, since each message may have included more than 1 recommended medical action, we defined a “screening success” as a case in which a member scheduled an appointment to at least 1 of their recommended screenings. Similarly, a “lab test success” was a case in which a member performed at least 1 of their recommended lab tests. Members who received a message that included a recommendation for a screening and a lab test were included in both groups for analysis purposes. Such members could count as a success in screenings or in lab tests or in both, depending on their actions. Following the criteria set by MHS, the time frame considered for measuring a success was 14 days from the last contact date for screenings, 30 days for the FOBT, and 60 days for pneumococcal vaccination (setting different time frames had no effect on the qualitative results and conclusions).

The time frame that we considered for opening a message was 45 days from the initial contact date. (Since the final reminder was sent roughly 4 weeks after the first message, this time frame ensured that we essentially examined all members who made the minimal effort to respond to the messages by opening at least 1 of them.) A text message was considered opened if a member clicked the link that appeared in the text message that redirected the member to the landing page with the content of the invitation message (an email was considered opened if the member viewed the body of the email).

### Covariates

In addition to the type of treatment (control, and 5 more treatment arms), we used the following covariates in our analysis:

- Gender: male or female.
- Age group: 50-54, 55-59, 60-64, 65-69, and 70-74 years.
- Socioeconomic status (SES): an index that is constructed by the Israel Central Bureau of Statistics (CBS), which incorporates information at the household level (education, income, employment, etc), alongside information regarding the geographical place of residence of the household and its local municipality. The index ranges from 1 to 10, where 1 reflects the weakest status and 10 the highest.
- Existing medical conditions (3 distinct covariates): indicating a previous history of (1) cardiac illness, (2) diabetes, or (3) a chronic condition of other type, such as being diagnosed with other chronic illnesses or receiving medication over a long duration of time.
- History of previous checkups: indicating whether the patient underwent any of the screenings or lab tests in the past.
- Media type: indicating whether the patient received an email message or an SMS text message.
- Periphery: indicating whether the patient resides in a geographically peripheral area (ie, based on the patient’s residence area relative to the central district of Israel).

### Statistical Analysis

We examined the effectiveness of the different framings for members who opened 1 of their messages, including reminders. To test the significance and effect of the treatment groups on either screenings or lab tests, we used 2 logistic regression models, respectively. The dependent variable was a dummy variable that received 1 if that observation was a success and 0 otherwise. The independent variables of interest were the treatment dummies. Our full set of covariates was added to the logistic regressions as control variables. We also tested for independence of the compliance rates versus association with each of the groups using a chi-square test (ie, independence test). In addition, we reported 95% CIs (via the proportion test) for the compliance rates with screenings and lab tests for the control and treatment groups.

We explored the relationship between the SES and email availability, as well as the relationship between the SES and message-opening rates, using visualization and computation of correlation coefficients. Logistic regression was then run to examine whether the effect of sending a message by email on the message-opening rate was positive even when the SES was controlled for. The dependent variable was a dummy variable that received 1 if the member opened the message and 0 otherwise. The independent variables of interest were media channels (email received 1, and text message received 0) and the SES.

We compared the opening rates of messages (proportion of email messages read vs proportion of text message link clicks) using a 2-sided proportion test. We also compared the proportion of test compliance, given message opening (again, using a 2-sided proportion test). These comparisons were performed for various subgroups of the population (ie, controlling for demographic characteristics).

We used 2-sided proportion tests (95% CI) to compare the opening rates of short-subject-line messages versus long-subject-line messages.

### Ethical Considerations

This research was approved by the Helsinki Committee of MHS (study number 0099-20-MHS) and by the Ethical Research and the Protection of Human Participants Committee, Faculty of Social Sciences, University of Haifa (approval number 369/21). Approvals included a waiver of consent. Identifying information was not shared with the researchers (ie, the researchers received anonymized data). No compensation was paid to participants.

## Results

### Sample Characteristics

Table 1 shows the demographic characteristics of our sample across treatments. (The table includes only members for whom we had all demographic variables and the SES, for a total of 112,802 members.) The treatment arms were well balanced on all observable characteristics, which included gender, age group, the SES, and existing medical conditions. Note that the age of the target group in the study and the broad definition of the “existing medical conditions” variable explain the high percentages of members who were classified as having a chronic condition.

**Table 1:** Demographics of the study sample by version.

Characteristics		Control (n=20,912, 18.5%), %	Gains (n=20,351, 18.0%), %	Losses (n=15,129, 13.4%), %	Recommendation (n=20,544, 18.2%), %	Implementation intentions (n=20,651, 18.3%), %	Empowerment (n=15,215, 13.5%), %	Total members (N=112,802), n (%)
**Gender**								
	Male	41.2	42.6	42.3	42.7	42.1	42.3	47,571 (42.2)
	Female	58.8	57.4	57.7	57.3	57.9	57.7	65,231 (57.8)
**Age (years)**								
	50-55	45.7	44.9	40.8	43.8	45.7	41.2	49,559 (43.9)
	55-60	15.2	15.0	16.6	15.3	14.8	16.1	17,364 (15.4)
	60-65	16.6	16.3	16.7	17.1	17.0	17.1	18,947 (16.8)
	65-70	12.5	13.0	13.8	12.8	12.2	13.8	14,605 (12.9)
	70-74	10.0	10.8	12.1	11.0	10.3	11.8	12,327 (10.9)
**SES^a^**								
	1	0.3	0.3	0.4	0.3	0.2	0.3	313 (0.3)
	2	1.5	1.7	2.3	1.7	1.7	2.0	2020 (1.8)
	3	7.4	7.5	8.1	7.4	7.8	8.1	8657 (7.7)
	4	9.9	10.2	10.5	9.7	9.8	11.2	11,465 (10.2)
	5	15.5	15.9	16.9	16.3	15.9	16.7	18,189 (16.1)
	6	19.6	18.7	19.4	19.3	18.9	18.8	21,551 (19.1)
	7	17.0	17.5	16.1	16.9	17.1	16.0	18,987 (16.8)
	8	14.6	14.8	13.5	14.9	14.9	13.7	16,329 (14.5)
	9	11.0	10.5	9.9	10.5	10.6	10.4	11,841 (10.5)
	10	3.2	3.0	3.0	3.1	3.1	2.9	3450 (3.1)
**Medical condition**								
	Diabetes	14.5	15.1	16.3	15.6	15.1	16.5	17,383 (15.4)
	Cardiovascular disease	6.8	7.3	7.6	7.2	7.2	7.8	8201 (7.3)
	Chronic illness	62.2	61.6	63.9	61.8	61.6	63.8	70,343 (62.4)

^a^SES: socioeconomic status. This is an index defined by the Central Bureau of Statistics (CBS), ranging from 1 to 10, where 1 reflects the weakest status and 10 the highest.

### Main Findings

Approximately 60% of the 113,048 participants in the study, totaling 67,772 MHS members, opened 1 of their messages within the 45-day time frame.

The first 2 columns in Table 2 report success rates (conditional on opening 1 of the messages) by treatment arm. There were no significant differences in success rates across treatments for screenings and for lab tests. Chi-squared tests did not allow us to reject the null hypothesis that compliance rates are independent of treatments (*P*=.37 for screenings and *P*=.88 for lab tests). The last 2 columns of the table relate to the message’s subject-line length (ie, number of words) and the respective message’s opening rate.

Table 3 includes logistic regressions with our full set of covariates added as controls (members who had missing values for any of the covariates were omitted from the regressions). Our null result is reflected in the table, as none of the framing versions had a significant effect on success rates. This null result held when we ran the same type of logistic regressions with interactions between the treatment variables and each of our controls (given the large number of interactions, we did not report the results of these regressions in the paper, but they are available upon request).

**Table 2:** Compliance rates by group (treatment or control).

Treatment	Compliance rate, total n (%); 95% CI		Subject-line length (words)	Message-opening rate, total n (%); 95% CI
	Screenings	Lab tests		
Control	5075 (14.1); 13.1%-15.1%	9581 (23.6); 22.7%-24.4%	8	20,959 (63.4); 62.7%-64.1%
Gains	4416 (13.9); 12.9%-15.0%	8687 (23.3); 22.4%-24.2%	15	20,393 (58.6); 57.9%-59.2%
Losses	3122 (12.9); 11.8%-14.1%	6027 (23.5); 22.4%-24.6%	14	15,165 (55.3); 54.5%-56.1%
Recommendation	4890 (13.1); 12.1%-14.1%	9357 (24); 23.2%-24.9%	11	20,584 (63.2); 62.6%-63.9%
Implementation intentions	4854 (14.1); 13.2%-15.2%	9015 (23.4); 22.6%-24.3%	16	20,701 (61.1); 60.5%-61.8%
Empowerment	3153 (14.1); 13.0%-15.4%	6133 (23.8); 22.7%-24.9%	14	15,246 (55.7); 54.9%-56.5%

**Table 3:** Two logistic regression models (for scheduling screenings and performing lab tests). For brevity, the following, statistically insignificant variables were omitted from the table (but were included in the model): gender, cardiovascular disease history, and diabetes history.

Variable		Dependent variable (success)	
		Screenings	Lab tests
**Independent variables, coefficient (SE)**			
	Constant	–2.325^a^ (0.099)	–1.434^a^ (0.058)
	Gains	0.005 (0.063)	–0.011 (0.037)
	Losses	–0.078 (0.07)	–0.055 (0.041)
	Recommendation	–0.073 (0.062)	0.027 (0.036)
	Implementation intentions	0.019 (0.061)	–0.003 (0.037)
	Empowerment	0.024 (0.069)	–0.034 (0.041)
	Conducted past checkup(s)	0.433^a^ (0.057)	0.262^a^ (0.028)
	Media channel (text message)	0.145^a^ (0.042)	0.159^a^ (0.025)
	Age 55-60 years	–0.128^b^ (0.058)	0.168^a^ (0.034)
	Age 60-65 years	–0.326^a^ (0.059)	0.153^a^ (0.033)
	Age 65-70 years	–0.324^a^ (0.065)	0.221^a^ (0.036)
	Age 70-74 years	–0.252^a^ (0.062)	0.44^a^ (0.039)
	SES^c^	0.026^b^ (0.011)	–0.032^a^ (0.006)
	Periphery	0.082 (0.072)	0.14^a^ (0.038)
	Chronic illness	0.105^b^ (0.048)	0.156^a^ (0.027)
Observations, n		22,992	44,052
Log likelihood		–9162	–23,937
Akaike information criteria		18,361	47,911

^a^*P*<.01.

^b^*P*<.05.

^c^SES: socioeconomic status. This is an index defined by the Central Bureau of Statistics (CBS), ranging from 1 to 10, where 1 reflects the weakest status and 10 the highest.

### Effectiveness by Media Channel

We compared the effectiveness of the media channels through which messages were sent (69,777, 61.7%, members received emails, and 43,271, 38.3%, members received text messages). Table 4 shows that these groups differed in terms of their demographic characteristics: Members who received text messages were older on average, had a lower SES, and were more likely to be males. More details are provided in the *Limitations* section. Therefore, we compared the performance of the different media channels within subgroups of the population with similar demographic characteristics.

Table 5 provides the opening rates of email messages, text messages, sample sizes, and the *P* value comparing the 2 rates for subgroups of the population with similar demographics. It is evident that text messages were disregarded more often than emails for every subgroup.

Table 6 provides for the same subgroups a comparison of the compliance rates of emails and text messages, given that a message was opened (rates, sample sizes, and *P* values). In most subgroups examined, and overall, text messages exhibited a slightly higher (but significant) conditional-compliance rate.

Table 7 shows the overall effectiveness of media channels (ie, the overall unconditional success rates of emails vs text messages). Email messages outperform text messages in all subgroups and overall.

**Table 4:** Demographics of the sample by media channel allocation.

Characteristic		Email (n=69,777), %	SMS text message (n=43,271), %
**Gender**			
	Male	40*.*8	44*.*4
	Female	59*.*2	55*.*6
**Age (years)**			
	50-55	47*.*1	38*.*8
	55-60	15*.*1	15*.*8
	60-65	16*.*1	17*.*9
	65-70	12*.*0	14*.*5
	70-74	9*.*7	12*.*9
**SES^a^**			
	1	0	0*.*7
	2	0*.*7	3*.*6
	3	4*.*9	12*.*2
	4	7*.*2	15*.*0
	5	13*.*5	20*.*3
	6	18*.*9	19*.*4
	7	19*.*1	13*.*1
	8	18*.*0	8*.*8
	9	13*.*6	5*.*4
	10	4*.*1	1*.*5

^a^SES: socioeconomic status. This is an index defined by the Central Bureau of Statistics (CBS), ranging from 1 to 10, where 1 reflects the weakest status and 10 the highest.

**Table 5:** Opening rates, sample size, and *P* values by media channel for demographic subgroups.

Characteristic			Message opening, %			Sample size, n				*P* value
		Email		SMS		Email	SMS			
**Age (years)**										
	50-55	65*.*3		50*.*2		32,836	16,801		<.001	
	55-60	64*.*5		50*.*9		10,551	6846		<.001	
	60-65	66*.*5		51*.*2		11,220	7758		<.001	
	65-70	67*.*6		51*.*3		8356	6271		<.001	
	70-74	67*.*7		48*.*3		6773	5570		<.001	
**SES^a^**										
	1-3	59*.*5		39*.*6		3897	7093		<.001	
	4-6	62*.*6		49*.*3		27,563	23,642		<.001	
	7-10	68*.*9		58*.*5		38,174	12,433		<.001	
**Gender**										
	Female	65*.*5		53*.*2		41,267	24,051		<.001	
	Male	66*.*4		46*.*9		28,469	19,195		<.001	
Overall			65*.*9		50*.*4	69,777		43,271		<.001

^a^SES: socioeconomic status. This is an index defined by the Central Bureau of Statistics (CBS), ranging from 1 to 10, where 1 reflects the weakest status and 10 the highest.

**Table 6:** Effectiveness of the media channel conditional on message opening for demographic subgroups.

Characteristic		Compliance rates, given message opening, %			Sample size, n		*P* value	
		Email	SMS	Email		SMS		
**Age (years)**								
	50-55	16*.*9	16*.*8	21,431		8436	*.*83	
	55-60	19*.*7	21*.*7	6803		3483	*.*01	
	60-65	18*.*1	21*.*5	7459		3970	<.001	
	65-70	19*.*1	22*.*0	5646		3216	*.*001	
	70-74	21*.*0	24*.*5	4587		2688	<*.*001	
**SES^a^**								
	1-3	18*.*7	20*.*5	2319		2808	*.*11	
	4-6	18*.*9	20*.*3	17,257		11,662	*.*003	
	7-10	17*.*7	19*.*7	26,284		7278	<*.*001	
**Gender**								
	Female	15*.*8	17*.*5	27,023		12,783	<.001	
	Male	21*.*6	23*.*9	18,903		9010	<.001	
Overall		18*.*2	20*.*2	45,961		21,811	<.001	

^a^SES: socioeconomic status. This is an index defined by the Central Bureau of Statistics (CBS), ranging from 1 to 10, where 1 reflects the weakest status and 10 the highest.

**Table 7:** Overall effectiveness (unconditional) of media channels for demographic subgroups.

Characteristic		Success rate, %		Sample size, n			*P* value
		Email	SMS	Email	SMS		
**Age (years)**							
	50-55	11*.*0	8*.*4	32,836	16,801	<*.*001	
	55-60	12*.*7	11*.*1	10,551	6846	*.*001	
	60-65	12*.*0	11*.*0	11,220	7758	*.*03	
	65-70	12*.*9	11*.*3	8356	6271	*.*003	
	70-74	14*.*2	11*.*8	6773	5570	<*.*001	
**SES^a^**							
	1-3	11*.*1	8*.*1	3897	7093	<*.*001	
	4-6	11*.*8	10*.*0	27,563	23,642	<*.*001	
	7-10	12*.*2	11*.*5	38,174	12,433	*.*04	
**Gender**							
	Female	10*.*4	9*.*3	41,267	24,051	<*.*001	
	Male	14*.*3	11*.*2	28,469	19,195	<*.*001	
Overall		12*.*0	10.2	69,777	43,271	<*.*001	

^a^SES: socioeconomic status. This is an index defined by the Central Bureau of Statistics (CBS), ranging from 1 to 10, where 1 reflects the weakest status and 10 the highest.

**Figure 1:**
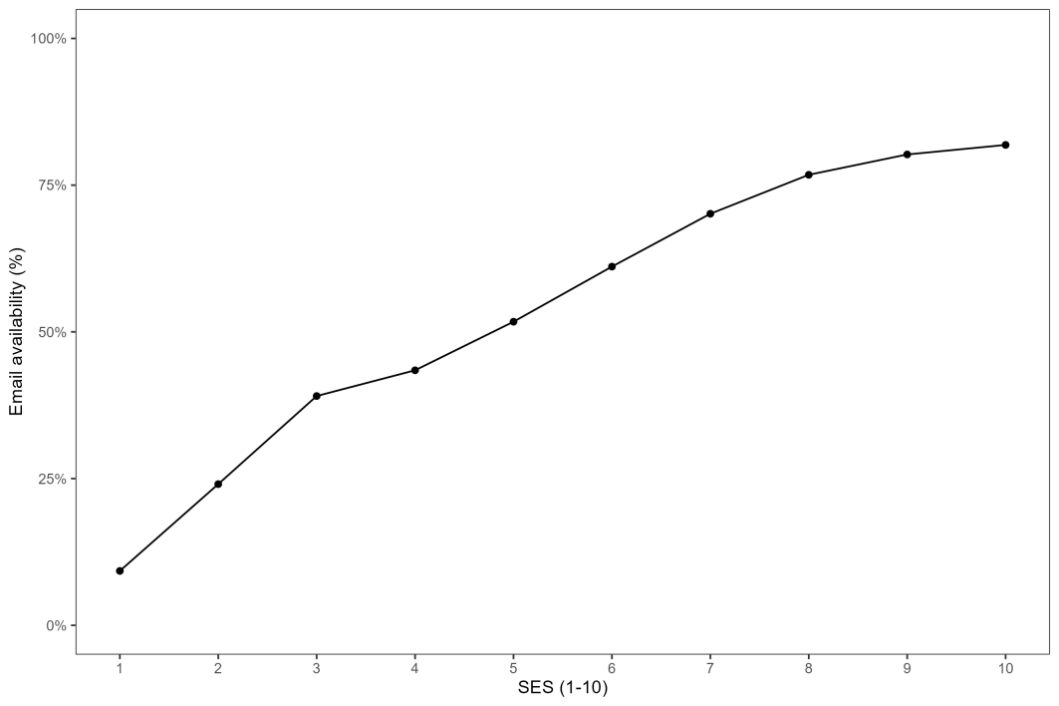
Email availability as a function of socioeconomic status (SES).

**Figure 2:**
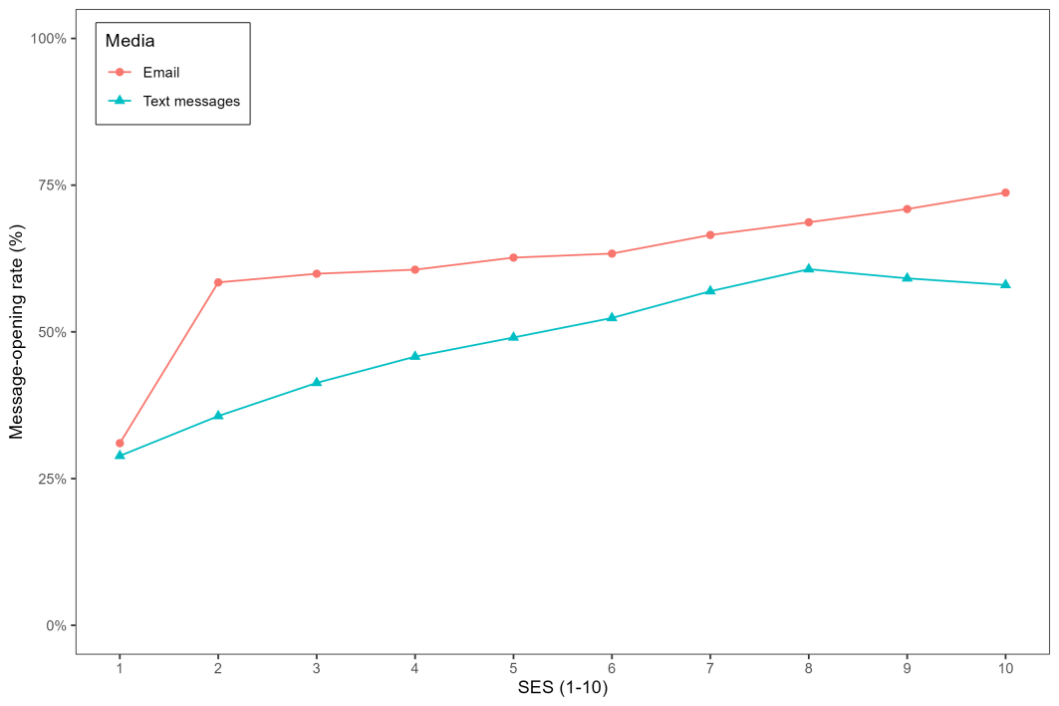
Message-opening rates as a function of the socioeconomic status (SES) for email and text messages (SMS).

We now provide results concerning the robustness of the findings reported in Table 5, according to which emails were opened more frequently than text messages. First, Figure 1 shows a strong positive relationship between email availability and the SES (correlation coefficient=0.298, *P*<.001). Next, Figure 2 visualizes a positive relationship between the message-opening rate and the SES for both media channels (correlation coefficient=0.079 and 0.13 for emails and text messages, respectively; *P*<.001 in both cases). Moreover, Figure 2 shows that emails had higher message-opening rates compared to text messages for each SES.

In Table 8, we report the results of regressing, in a logistic model, the message-opening rates on the media channel being email and the SES. The email media channel positively affected the message-opening rates even when the SES (which had a positive effect of its own) was controlled for. Table 8 reports this result without controls and while controlling for the entire set of covariates.

**Table 8:** Two logistic regression models for message-opening rates, with no controls and controlling for all available demographic characteristics and chronic conditions.

Variable		Dependent variable (message opening)			
		Without controls	Full set of controls		
**Independent variable** **, coefficient (SE)**					
	Constant	–0.596^a^ (0.021)	–0.642^a^ (0.028)		
	SES^b^	0.112^a^ (0.003)	0.112^a^ (0.004)		
	Media channel (email)	0.516^a^ (0.013)	0.513^a^ (0.014)		
	Gender (male)	—^c^	–0.034^d^ (0.014)		
	Age 55-60 years	—	0.004 (0.02)		
	Age 60-65 years	—	0.059^a^ (0.019)		
	Age 65-70 years	—	0.089^a^ (0.021)		
	Age 70-74 years	—	0.024 (0.023)		
	Cardiovascular disease	—	–0.019 (0.025)		
	Diabetes	—	–0.111^a^ (0.018)		
	Chronic illness	—	0.037^d^ (0.016)		
	Periphery	—	–0.018 (0.022)		
	Conducted past checkup(s)	—	0.058^a^ (0.017)		
Observations, n		112,802	101,675		
Log likelihood		–74,063	–66,604		
Akaike information criteria		148,133	133,234		

^a^*P*<.01.

^b^SES: socioeconomic status. This is an index defined by the Central Bureau of Statistics (CBS), ranging from 1 to 10, where 1 reflects the weakest status and 10 the highest.

^c^These variables were not included in this regression model.

^d^*P*<.05.

### Message-Opening Rates and Subject-Line Length

**Figure 3:**
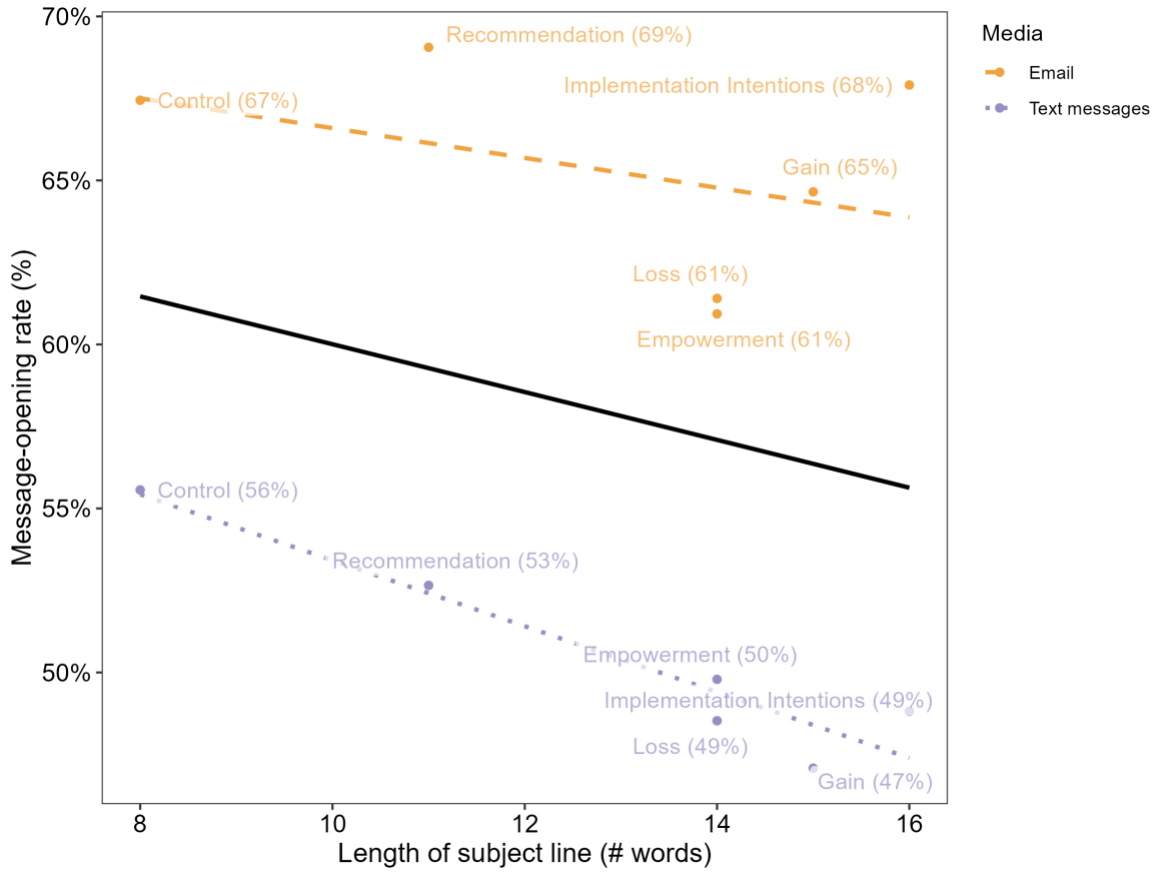
Message-opening rates as a function of the subject-line length, with a linear regression fit for email (dashed line), text messages (dotted line), and overall (solid line).

Figure 3 shows the relationship between message-opening rates and the subject-line length for the different media channels and overall (see Table 2 for 95% CIs). As the length of the subject line increased, the message-opening rate decreased. For emails, the average opening rate of the 2 messages with the shortest subject lines (8 and 11 words) was 68.2% and the average opening rate of the 2 messages with the longest subject line (15 and 16 words) was 66.3%. For text messages, these rates were 54.1% and 48%, respectively (both differences were significant, with *P*<.001).

## Discussion

### Principal Findings

Our main findings do not allow rejecting the null hypothesis according to which uptake rates of medical checkups are independent of the message framing. Compliance rates (whether appointment scheduling or actual performance) are relatively similar across treatments, and this finding holds even after controlling for different demographic and health-related covariates.

We identified several variables that have a significant effect on compliance: conducting a medical procedure in the past increases the chances of compliance, as expected. Text messages seem to increase uptake rates, conditional on opening the message—a finding that is also robust when examining subgroups with similar demographic characteristics. It is interesting to note that age has a significant effect on success rates but the direction of the effect changes depending on the type of checkups (the reference age group in the regressions was 50-55 years).

The reason for these directional effects is likely the following: Older members may find it more difficult to schedule appointments through the online interface—hence the negative coefficients for the older age groups when we measured screening success. By contrast, lab tests only require a visit to the clinic and no appointments. Older members tend to visit their local clinic more regularly than younger members, which leads to the observed positive effect of age on lab test success. The variable for the SES also showed opposing effects, depending on the type of checkup. As one might expect, appointments made through the online interface increased with higher SES. By contrast, a higher SES leads to lower levels of performance of lab tests. Although we cannot delineate a clear explanation for this finding, it may be that, similar to older adults, individuals of a lower SES tend to visit their local clinics more often than those with a higher SES and therefore have higher performance rates of lab tests.

Regarding the effectiveness of media channels, we conducted this analysis for subgroups of the population with similar demographics, since there was no random assignment to media channels and the 2 groups apparently had different characteristics. For message-opening rates, we found highly significant differences favoring emails for every subgroup (Table 5).

Within each media method, we found that shorter subject lines increase the likelihood of opening messages. Given our main finding of no effect of framing on appointment scheduling/actual performance, we believe that this pattern suggests that shorter subject lines, rather than the subject line’s content, increase the likelihood of opening a message.

Conditional on message opening, we observed that text messages had a significant edge for 8 of 10 subgroups, as shown in Table 6. The overall effectiveness (ie, unconditional success rate) of each channel showed that emails outperform text messages and significantly so for all subgroups (Table 7). We concluded that the high message-opening rates for emails compared to text messages is the driving force behind this media channel’s increased success in our study.

The observation that text messages are disregarded more often than emails may be because text messages are considered more intrusive than emails. However, once opened, members are more likely to actually make an appointment or take the recommended medical action in the case of a text message than when reading through an email (Table 6). It may be that those who are willing to open a text message rather than disregard it have a more positive attitude toward health care recommendations than those willing to read through an email.

There was a positive relationship between the message-opening rate and the SES for both media channels. Moreover, emails had higher message-opening rates than text messages for each SES. These observations suggest that message-opening rates are positively affected by both the SES and the media channel being email.

These findings may be valuable for future public health campaigns as they provide information about the potential of different media channels to fulfil their designated purpose. However, the lack of random assignment into the different media channels prevents us from drawing firm conclusions on this matter. More research needs to be conducted (specifically with random assignment to media channels) in order to substantiate these findings.

### Relation to the Literature and Contribution

Two of the framings used in our study, gains and losses, which are based on prospect theory [12-14], are the most commonly studied in the literature on framing of health-related communication. Dozens of studies have examined their effectiveness in nudging individuals toward desired healthy behaviors, including early-detection screening tests for different types of cancer. In the past 3 decades, evidence has amounted and has shown mixed results with respect to the empirical validity of these findings (see Refs. [5,15,40,41] for systematic reviews). As a result, conceptual concerns regarding their use in health-related behavior change have been raised [16]. Furthermore, it has recently been suggested that there is not much difference between the effectiveness of the 2 types of messages as far as persuasiveness is concerned [42], even when accounting for different moderating factors. Our study fits right into this discussion and allows testing this paradigm, and the other types of message framings that we have taken from the literature (implementation intentions, recommendation, and empowerment), in a large-scale field study. As mentioned before, we found no evidence that these types of framings are more effective in increasing compliance rates with medical checkups than a standard neutrally framed invitation message. These results suggest that new framings may need to be devised to attract individuals’ attention to important health recommendations.

Our 2 additional findings indicate that more research should be put into the exploration of the effect of the method of delivery of digital messages on uptake rates of medical checkups. The literature on the effect of the delivery medium on preventive health care outcomes has, thus far, been unable to clearly point toward a specific medium that is superior to the rest [43]. Findings to date suggest that all types of mediums are generally successful at encouraging behavior change, but there is no clear emerging pattern linking the type of medium and the type of message to adherence with specific health behaviors [33,34,36,44]. Moreover, a recent review of the potential effects of text messages in promoting health behavior change has called for large-scale implementations of text messages in order to better assess their pros and cons [45]. Our study contributes to this literature as we found that each communication medium in our study surpasses the other along a different dimension:

- Message-opening rates: The percentage of email recipients who opened their emails surpassed the percentage of SMS text message recipients who clicked the enclosed link in the text message.
- Compliance conditional on message opening: Among those who opened their invitation message (email or SMS text message), we observed higher compliance rates (ie, higher success rates) in SMS text message recipients compared to email recipients.

Given the unique strengths of each communication channel, our goal was to evaluate and compare their overall effectiveness (specifically, to measure compliance rates among recipients of each communication type). Our findings showed higher compliance rates among email recipients in comparison to the compliance rates of those receiving SMS text messages. Consequently, we concluded that the increased rates of email-opening rates elevate the overall compliance beyond what is observed for SMS text messages.

We think that examining these 2 media channels more carefully could be an exciting topic for future research. It also entails clear policy implications regarding tailoring the preferred media channel and combining the channels in specific campaigns. Messages that call for action and are expected (in advance) to have high opening rates due to their high relevance (eg, COVID 19–related messages during the recent pandemic) may benefit from text messages. However, messages that refer to routine checkups and ongoing recommendations, as those that appeared in our study, may benefit from being sent by email, as they may be opened more frequently and consequently will receive more attention than if they are delivered by SMS.

Regarding the subject-line length, it has received increasing attention in recent marketing literature [37-39], but we are unaware of any studies that examine this topic in relation to health behavior. Our suggestive finding that the subject-line length is negatively correlated with message-opening rates may trigger future research on the topic. Designing a field experiment that examines the effect of additional words/characters in messages’ subject lines on message-opening rates and the overall compliance of health-related messages may have important implications for the success of future health behavior campaigns in reaching their target audience.

### Limitations

One limitation of our study is that there was no random assignment to media channels. Members with no valid email address in the registry of MHS received a text message. To deal with this limitation, we conducted the relevant analysis for subgroups with similar demographic characteristics. However, it is possible that there are also unobserved characteristics that differ across the 2 groups. This prevented us from drawing firm conclusions regarding the effectiveness of the different media channels. More research needs to be conducted (specifically with random assignment to media channels) in order to substantiate these findings. Another potential limitation, as in many text message interventions, is the lack of control over whether members who opened their message actually read it. It is possible that some skim through their messages without paying much attention to the content (see Ref. [46] for a recent pilot study regarding patients’ reading rates of emails sent by their physicians). The random assignment to different groups should attenuate the potential effects of this limitation. To further address this issue and strengthen the effect of the framing, we included the main theme of each framing, not only in the body of the message, but also in the subject line. However, one should acknowledge the fact that in the era of digital messaging, limited attention of recipients is likely and should be considered. In this respect, our field study realistically mirrors the potential difficulties that future digital campaigns are likely to encounter. Specifically, it may be more difficult to generate a specific state of mind by framing in the digital era compared to the traditional communication methods (eg, brochures, fliers, post).

### Policy Implications

Our findings have important policy implications since message framings are frequently used by health care providers to increase individuals’ compliance rates with health recommendations. Taking a broader perspective, it raises questions regarding the ability of specific textual nudges to increase the public’s adherence and compliance rates with different types of government regulations and professional recommendations. It has been recently argued that accounting for publication bias, these nudges may have a small or no effect in different contexts [47,48]. Our findings add to this ongoing discussion and may suggest that there is room for novel framings or other types of behavioral nudges in order to increase compliance rates with preventive care recommendations. It also sheds light on the seemingly large potential effect of the choice of media through which messages are delivered and the subject-line length. These choices may seem of low consequence at first glance, but our suggestive findings imply otherwise. Future research that carefully examines these topics on a large scale may prove useful for the success of future digital health care campaigns.

### Conclusion

This study provides evidence regarding the usefulness of well-known framings from health communication research in a large-scale digital field experiment held in Israel. Our main finding is that compliance with recommendations is not affected by the types of framings we used. We believe that this evidence, coming from a wide outreach campaign, shows that digital messaging may require new framings in order to enhance compliance in the digital era. We also report suggestive evidence on significant differences between the effectiveness of the media channels used in our study (email and SMS) and on the effect of subject-line length on message-opening rates.

## Appendix

Multimedia Appendix 1 Message contents.

Multimedia Appendix 2 Data cleaning.

## Abbreviations

CBS: Central Bureau of Statistics
FOBT: fecal occult blood test
HPV: human papillomavirus
MHS: Maccabi Healthcare Services
SES: socioeconomic status

## Data Availability

The code will be made available on a public GitHub repository. The pre-registration is available through the American Economic Association’s (AEA) randomized controlled trial (RCT) registry [49]. The individual-level data sets used in this study are not publicly available, as we do not have legal permission to publish them due to Maccabi Healthcare Services (MHS) policy. MHS considers these to be sensitive health data that contain individual-level information regarding health. These type of data are not made available even if they are deidentified (as it may still be possible to reidentify patients from such data). To keep transparency as high as possible, given this limitation, we have posted our code for the analysis to a public GitHub repository. In addition, some of the tables in the paper provide aggregated data for different variables (eg, Tables 1, 4-7). In special cases of interest, a request may be sent to the corresponding author, who will submit a special inquiry to MHS for partial or full access to the data. Access may still be denied as the decision will be made by MHS in compliance with its data sharing policy.

## References

[1] Abroms LC, Boal AL, Simmens SJ, Mendel JA, Windsor RA (2014). A randomized trial of Text2Quit: a text messaging program for smoking cessation. Am J Prev Med.

[2] Free C, Knight R, Robertson S, Whittaker R, Edwards P, Zhou W, Rodgers A, Cairns J, Kenward MG, Roberts I (2011). Smoking cessation support delivered via mobile phone text messaging (txt2stop): a single-blind, randomised trial. Lancet.

[3] Kock L, Brown J, Hiscock R, Tattan-Birch H, Smith C, Shahab L (2019). Individual-level behavioural smoking cessation interventions tailored for disadvantaged socioeconomic position: a systematic review and meta-regression. Lancet Public Health.

[4] Finney LJ, Iannotti RJ (2002). Message framing and mammography screening: a theory-driven intervention. Behav Med.

[5] Gallagher KM, Updegraff JA (2012). Health message framing effects on attitudes, intentions, and behavior: a meta-analytic review. Ann Behav Med.

[6] Buttenheim A, Milkman KL, Duckworth AL, Gromet DM, Patel M, Chapman G (2022). Effects of ownership text message wording and reminders on receipt of an influenza vaccination: a randomized clinical trial. JAMA Netw Open.

[7] Yokum D, Lauffenburger JC, Ghazinouri R, Choudhry NK (2018). Letters designed with behavioural science increase influenza vaccination in Medicare beneficiaries. Nat Hum Behav.

[8] Milkman KL, Patel MS, Gandhi L, Graci H, Gromet D, Ho QDH, Kay J, Lee T, Akinola M, Beshears J, Bogard J, Buttenheim A, Chabris C, Chapman GB, Choi JJ, Dai H, Fox CR, Goren A, Hilchey M, Hmurovic J, John L, Karlan D, Kim M, Laibson DI, Lamberton C, Madrian BC, Meyer MN, Modanu M, Nam J, Rogers T, Rondina R, Saccardo S, Shermohammed M, Soman D, Sparks J, Warren C, Weber M, Berman R, Evans C, Snider C, Tsukayama E, Van den Bulte C, Volpp K, Duckworth A (2021). A megastudy of text-based nudges encouraging patients to get vaccinated at an upcoming doctor's appointment. Proc Natl Acad Sci U S A.

[9] Bokemper SE, Gerber AS, Omer SB, Huber GA (2021). Persuading US White evangelicals to vaccinate for COVID-19: testing message effectiveness in fall 2020 and spring 2021. Proc Natl Acad Sci U S A.

[10] Dai H, Saccardo S, Han MA, Roh L, Raja N, Vangala S, Modi H, Pandya S, Sloyan M, Croymans DM (2021). Behavioural nudges increase COVID-19 vaccinations. Nature.

[11] Mehta SJ, Mallozzi C, Shaw PA, Reitz C, McDonald C, Vandertuyn M, Balachandran M, Kopinsky M, Sevinc C, Johnson A, Ward R, Park S, Snider CK, Rosin R, Asch DA (2022). Effect of text messaging and behavioral interventions on COVID-19 vaccination uptake: a randomized clinical trial. JAMA Netw Open.

[12] Kahneman D, Tversky A (2013). Prospect theory: an analysis of decision under risk. Handbook of the Fundamentals of Financial Decision Making.

[13] Tversky A, Kahneman D (1991). Loss aversion in riskless choice: a reference-dependent model. Q J Econ.

[14] Tversky A, Kahneman D (1981). The framing of decisions and the psychology of choice. Science.

[15] O?Keefe D, Jensen J (2009). The relative persuasiveness of gain-framed and loss-framed messages for encouraging disease detection behaviors: a meta-analytic review. J Commun.

[16] Van't Riet J, Cox AD, Cox D, Zimet GD, De Bruijn G, Van den Putte B, De Vries H, Werrij MQ, Ruiter RAC (2016). Does perceived risk influence the effects of message framing? Revisiting the link between prospect theory and message framing. Health Psychol Rev.

[17] Bazargan M, Bazargan SH, Calderón J L, Husaini BA, Baker RS (2003). Mammography screening and breast self-examination among minority women in public housing projects: the impact of physician recommendation. Cell Mol Biol (Noisy-le-grand).

[18] Guerra CE, Schwartz JS, Armstrong K, Brown JS, Halbert CH, Shea JA (2007). Barriers of and facilitators to physician recommendation of colorectal cancer screening. J Gen Intern Med.

[19] Hudson SV, Ferrante JM, Ohman-Strickland P, Hahn KA, Shaw EK, Hemler J, Crabtree BF (2012). Physician recommendation and patient adherence for colorectal cancer screening. J Am Board Fam Med.

[20] Gollwitzer PM (1999). Implementation intentions: strong effects of simple plans. Am Psychol.

[21] Gollwitzer P, Sheeran P (2006). Implementation intentions and goal achievement: a meta‐analysis of effects and processes. Adv Exp Soc Psychol.

[22] Gollwitzer PM, Oettingen G (1998). The emergence and implementation of health goals. Psychol Health.

[23] Milkman KL, Beshears J, Choi JJ, Laibson D, Madrian BC (2011). Using implementation intentions prompts to enhance influenza vaccination rates. Proc Natl Acad Sci U S A.

[24] Neter E, Stein N, Barnett-Griness O, Rennert G, Hagoel L (2014). From the bench to public health: population-level implementation intentions in colorectal cancer screening. Am J Prev Med.

[25] Sheeran P, Orbell S (2000). Using implementation intentions to increase attendance for cervical cancer screening. Health Psychol.

[26] Lo SH, Good A, Sheeran P, Baio G, Rainbow S, Vart G, von Wagner C, Wardle J (2014). Preformulated implementation intentions to promote colorectal cancer screening: a cluster-randomized trial. Health Psychol.

[27] Luszczynska A, Durawa AB, Scholz U, Knoll N (2012). Empowerment beliefs and intention to uptake cervical cancer screening: three psychosocial mediating mechanisms. Women Health.

[28] Consedine NS, Horton D, Magai C, Kukafka R (2007). Breast screening in response to gain, loss, and empowerment framed messages among diverse, low-income women. J Health Care Poor Underserved.

[29] Cooper DP, Goldenberg JL, Arndt J (2011). Empowering the self: using the terror management health model to promote breast self-examination. Self Identity.

[30] Lim MSC, Hocking JS, Aitken CK, Fairley CK, Jordan L, Lewis JA, Hellard ME (2012). Impact of text and email messaging on the sexual health of young people: a randomised controlled trial. J Epidemiol Community Health.

[31] Armanasco AA, Miller YD, Fjeldsoe BS, Marshall AL (2017). Preventive health behavior change text message interventions: a meta-analysis. Am J Prev Med.

[32] Johnson-Mallard V, Darville G, Mercado R, Anderson-Lewis C, MacInnes J (2019). How health care providers can use digital health technologies to inform human papillomavirus (HPV) decision making and promote the HPV vaccine uptake among adolescents and young adults. Biores Open Access.

[33] Kulhánek A, Lukavska K, Gabrhelík R, Novák D, Burda V, Prokop J, Holter MTS, Brendryen H (2022). Comparing reminders sent via SMS text messaging and email for improving adherence to an electronic health program: randomized controlled trial. JMIR Mhealth Uhealth.

[34] Gram IT, Larbi D, Wangberg SC (2019). Comparing the efficacy of an identical, tailored smoking cessation intervention delivered by mobile text messaging versus email: randomized controlled trial. JMIR Mhealth Uhealth.

[35] Stead M, Angus K, Langley T, Katikireddi SV, Hinds K, Hilton S, Lewis S, Thomas J, Campbell M, Young B, Bauld L (2019). Mass media to communicate public health messages in six health topic areas: a systematic review and other reviews of the evidence. Public Health Res.

[36] Webb TL, Joseph J, Yardley L, Michie S (2010). Using the internet to promote health behavior change: a systematic review and meta-analysis of the impact of theoretical basis, use of behavior change techniques, and mode of delivery on efficacy. J Med Internet Res.

[37] Balakrishnan R, Parekh R (2014). Learning to predict subject-line opens for large-scale email marketing.

[38] Chaparro-Peláez J, Hernández-García Á, Lorente-Páramo Á-J (2022). May I have your attention, please? An investigation on opening effectiveness in e-mail marketing. Rev Manag Sci.

[39] Kumar A, Salo J (2016). Effects of link placements in email newsletters on their click-through rate. J Mark Commun.

[40] Akl E, Oxman A, Herrin J, Vist G, Terrenato I, Sperati F, Costiniuk C, Blank D, Schünemann H (2011). Framing of health information messages. Cochrane Database Syst Rev.

[41] Pope JP, Pelletier L, Guertin C (2018). Starting off on the best foot: a review of message framing and message tailoring, and recommendations for the comprehensive messaging strategy for sustained behavior change. Health Commun.

[42] O'Keefe DJ, Hoeken H (2021). Message design choices don’t make much difference to persuasiveness and can’t be counted on—not even when moderating conditions are specified. Front Psychol.

[43] Muench F, Baumel A (2017). More than a text message: dismantling digital triggers to curate behavior change in patient-centered health interventions. J Med Internet Res.

[44] De Leon E, Fuentes LW, Cohen JE (2014). Characterizing periodic messaging interventions across health behaviors and media: systematic review. J Med Internet Res.

[45] Willcox JC, Dobson R, Whittaker R (2019). Old-fashioned technology in the era of “bling”: is there a future for text messaging in health care?. J Med Internet Res.

[46] Reiter A, Tov EY, Hochberg I (2023). Do patients read emails from their physician containing tips on improving lifestyle habits? A pilot study. Int J Med Inform.

[47] DellaVigna S, Linos E (2022). RCTs to scale: comprehensive evidence from two nudge units. ECTA.

[48] Maier M, Bartoš F, Stanley TD, Shanks DR, Harris AJL, Wagenmakers E (2022). No evidence for nudging after adjusting for publication bias. Proc Natl Acad Sci U S A.

[49] Increasing cancer screening rates and vaccine uptake using digital messages based on insights from behavioral economics. AEA RCT Registry.

