# Supplementary material for: The Framing Effect of Digital Textual Messages on Uptake Rates of Medical Checkups: Field Study
Source: JMIR Public Health Surveill. 2024 Mar 6;10:e45379. doi: 10.2196/45379 (PMC10955408; doi:10.2196/45379)
Supplement: Multimedia Appendix 1 [file publichealth_v10i1e45379_app1.docx]

# Message Contents

Below are the translations to English of the contents of each of the original emails (that were sent out in Hebrew). The reminders (not reported below) are almost identical with minor differences in the wording of the subject line which were introduced in order to avoid repetitiveness. The specific words in each treatment that are different from the control appear in *italics*. In addition to the differences in the subject lines and contents, emails also included a pre-header which was almost identical to the subject line, appeared just below the subject line and reiterated the treatment's frame as shown below (text messages did not contain a pre-header).

For each frame, the content of the text message was identical to the subject line of the email except for the fact that it included a link that redirected members to a landing page. The content on the landing page was identical to the content of the email. Keep in mind that the results in the paper regarding the length of the subject lines, refer to the number of words in the original messages (in Hebrew).

All messages included links that redirected to more information on MHS’ website regarding the specific medical procedures that were recommended for the member. These links appeared in the email itself (if an email was used to contact the member) or on the landing page that members’ reached after clicking on the link in the text message (in case the member was contacted by SMS).

**Control**.

Subject line: Dear member, here are the checkups that are important for you to perform soon.

Pre-header: The checkups that will help you maintain a healthy life style.

Content: Here are the checkups that are recommended for your age group by the ministry of health. Peforming the checkups helps maintain a healthy life.

**Gains**.

Subject line: Dear member, *Early detection increases the chances of full recovery*. Here are the checkups that are important for you to perform soon.

Pre-header: The checkups that will help you maintain a healthy life style. *Early detection increases the range of potential treatments.*

Content: Here are the checkups that are recommended for your age group by the ministry of health. Performing the checkups helps maintain a healthy life. *Early detection of a medical condition increases the range of potential treatments and significantly increases the chances of full recovery*.

**Losses**.

Subject line: Dear member, *Late detection lowers the chances of full recovery*. Here are the checkups that are important for you to perform soon.

Pre-header: The checkups that will help you maintain a healthy life style. *Late detection decreases the range of potential treatments.*

Content: Here are the checkups that are recommended for your age group by the ministry of health. Performing the checkups helps maintain a healthy life. *Late detection of a medical condition lowers the range of potential treatments and significantly decreases the chances of full recovery*.

**Recommendation**.

Subject line: Dear member, here are the checkups *that the physicians at MHS recommend that* you perform soon.

Pre-header: The checkups that *according to the physicians at MHS* will help you maintain a healthy life style.

Content: Here are the checkups that are recommended for your age group by *the physicians of MHS and* the ministry of health. Performing the checkups helps maintain a healthy life.

**Implementation Intentions**.

Subject line: Dear member, *we made a follow up medical plan to take care of your health that includes* the checkups that are important for you to perform soon.

Pre-header: *Make an appointment and perform the checkups - the first stage in your follow up medical plan.*

Content: Here are the checkups that are recommended for your age group by the ministry of health *in order to assist you in maintaining* a healthy life. *After you perform the checkups, are the results reassuring? Great! We will contact you if future checkups are required to help you maintain a healthy life. Are the results not so reassuring? It’s good that you found out. Contact your physician to receive the information regarding the next steps*.

**Empowerment**.

Subject line: Dear member, *The responsibility for your health is in your hands*. Here are the checkups that are important for you to perform soon.

Pre-header: *The responsibility is in your hands.* The checkups that will help you maintain a healthy life style.

Content: Here are the checkups that are recommended for your age group by the ministry of health. *The responsibility for your health is in your hands*: Taking the tests helps maintain a healthy life.
