# Supplementary material for: The Framing Effect of Digital Textual Messages on Uptake Rates of Medical Checkups: Field Study
Source: JMIR Public Health Surveill. 2024 Mar 6;10:e45379. doi: 10.2196/45379 (PMC10955408; doi:10.2196/45379)
Supplement: Multimedia Appendix 2 [file publichealth_v10i1e45379_app2.docx]

# Data-Cleaning Procedure

The study began on July 29th, 2020, and ran up to December 6^th^, 2021. Note that MHS started sending out digital messages a year earlier with only one invitation message, which in the study became the control message. In our analysis we only included members who received their first invitation message after the study was initiated. The total number of such members was 129,070.

We removed members whose data showed inconsistencies according to the following steps:

1. 173 members received the version of the second or third reminder before the original invitation message.
2. 2 members appeared to have followed a recommendation given to them prior to the study’s initiation date.
3. 5,330 members received more messages than the amount designated by the campaign.
4. 484 members received messages through more than one media channel, e.g., original invitation by email and first reminder as a text message.
5. 10,017 members received messages and reminders that included multiple frames.
6. 16 members had no specified frame.

Following these steps, we were left with 113,048 members.
